# Gla Rich Protein (GRP) Mediates Vascular Smooth Muscle Cell (VSMC) Osteogenic Differentiation, Extracellular Vesicle (EV) Calcification Propensity, and Immunomodulatory Properties

**DOI:** 10.3390/ijms252212406

**Published:** 2024-11-19

**Authors:** Carla Viegas, Joana Carreira, Teresa M. Maia, Anjos L. Macedo, António P. Matos, José Neves, Dina Simes

**Affiliations:** 1 Centre of Marine Sciences (CCMAR/CIMAR LA), University of Algarve, 8005-139 Faro, Portugal; jscarreira@ualg.pt (J.C.); dsimes@ualg.pt (D.S.); 2 GenoGla Diagnostics, Centre of Marine Sciences (CCMAR), University of Algarve, 8005-139 Faro, Portugal; 3 VIB Center for Medical Biotechnology, Technologiepark-Zwijnaarde 75, 9052 Ghent, Belgium; teresa.maia@vib-ugent.be; 4 Department of Biomolecular Medicine, Ghent University, Technologiepark-Zwijnaarde 75, 9052 Ghent, Belgium; 5 VIB Proteomics Core, 9052 Ghent, Belgium; 6 UCIBIO, Department of Chemistry, and Associate Laboratory i4HB—Institute for Health and Bioeconomy, NOVA School of Science and Technology, Universidade NOVA de Lisboa, 2829-516 Caparica, Portugal; mdam@fct.unl.pt; 7 Egas Moniz Center for Interdisciplinary Research (CiiEM), Egas Moniz School of Health & Science, 2829-511 Caparica, Portugal; apamatos@gmail.com; 8 Service of Cardiothoracic Surgery, Santa Cruz Hospital, Centro Hospitalar de Lisboa Ocidental, 2790-134 Carnaxide, Portugal; josepedroneves@hotmail.com

**Keywords:** vascular calcification, extracellular vesicles, inflammation, gla rich protein

## Abstract

Vascular calcification (VC) is a complex process involving vascular smooth muscle cell (VSMC) osteogenic differentiation, inflammation, and extracellular vesicle (EV) calcification and communication networks. Gla rich protein (GRP) is a calcification inhibitor involved in most of these processes. However, the molecular mechanism of GRP in VC and the specific characteristics, cargo, and functionality of calcifying EVs require further elucidation. Here, we use a combination of human ex vivo aortic fragments and primary vascular smooth muscle cell (VSMC) models to obtain new information on GRP function in VC and EVs released by VSMCs. We demonstrate that GRP inhibits VSMC osteogenic differentiation through downregulation of bone-related proteins and upregulation of mineralization inhibitors, with decreased mineral crystallinity in EVs deposited into the tissue extracellular matrix (ECM). EVs isolated by ultracentrifugation at 30K and 100K from the cell media (CM) and deposited in the ECM from control (CTR) and mineralizing (MM) VSMCs were biochemically, physically, and proteomically characterized. Four different EV populations were identified with shared markers commonly present in all EVs but with unique protein cargo and specific molecular profiles. Comparative proteomics identified several regulated proteins specifically loaded into MM EV populations associated with multiple processes involved in VC. Functional analysis demonstrated that 30K and 100K ECM-MM EVs with higher calcium and lower GRP levels induced macrophage inflammation. Our findings reinforce the functional relevance of GRP in multiple VC processes and suggest that ECM EVs released under calcification stress function as a new signaling axis on the calcification–inflammation cycle.

## 1. Introduction

Vascular calcification (VC) is recognized as a strong predictor of cardiovascular morbidity and mortality, contributing to vessel stiffening, increased pulse pressure and left ventricular hypertrophy, atherosclerosis development, and plaque rupture, which reflect in clinical outcomes such as heart failure, myocardial infarction, stroke, coronary artery disease, and sudden cardiac death [1,2]. Chronic kidney disease (CKD) and atherosclerosis are the clinical conditions that most contribute to the development of VC at the intimal and medial layers of vessel walls [3].

Though our current knowledge on the molecular mechanisms of VC have increased considerably in recent years, many gaps still exist due to its multifactorial nature. Several interconnected pathological processes occur in complex environments, involving different cells and plastic cells capable of acquiring multiple phenotypes. Although the sequence of events and the interplay between molecular processes are still not completely elucidated, the dysregulation of endogenous calcification inhibitors, dedifferentiation of vascular smooth muscle cells (VSMCs), and inflammation and calcification prone to extracellular vesicles (EVs) are known as the master pathological processes involved in the initiation and progression of VC [4]. The loss of calcification inhibitors, increased proinflammatory mediators, and oxidative stress are known to be involved in the osteochondrogenic differentiation of VSMCs, characterized by increased expression of bone-related proteins and release of EVs with calcifying capacity [5]. These EVs are deficient in mineralization inhibitors and enriched in calcification prone factors and act as a first nidus for extracellular matrix (ECM) calcification [6,7,8]. Increased osteogenic factors and microcalcifications can in turn promote proinflammatory reactions in immune cells and VSMCs, while increased production of proinflammatory cytokines such as interleukin-1 beta (IL-1β) and tumor necrosis factor alpha (TNFα) promote increased expression of osteogenic proteins, further amplifying calcification and inflammation in a pathological cycle [9,10,11,12,13,14,15,16]. This positive feedback loop is of particular relevance in atherosclerosis, involving the interplay of multiple cells such as VSMCs, macrophages, and endothelial cells (ECs). In this complex environment, EVs have been shown as key players in inter- and intracellular communication networks. Extracellular vesicle is a collective term for the nanosized membrane-bound structures secreted by all human cell types into the extracellular space, containing bioactive cargoes such as proteins, RNA, lipids, and DNA, which can be uptaken by recipient cells, altering their phenotype [17]. EVs are classically classified into three subtypes based on size and biogenesis, with exosomes from endocytic origin smaller than 150 nm, microvesicles shed from the plasma membrane ranging from 100 to 1000 nm, and apoptotic bodies shed from dying cells ranging from 100 nm to 5 mm [17,18]. However, specific biogenesis classification is challenging due to overlapping physical and molecular characteristics and a lack of consensus markers [18,19]. In VC, several in vivo and in vitro studies have shown the crucial role of EVs in initiating and propagating microcalcifications as well as an important signaling axis between cells. Inflammation driven by activated macrophages has been shown to directly contribute to ECM microcalcifications through the release of EVs with high calcification potential [20,21,22,23]. Also, EVs released by inflamed ECs modulate inflammatory responses in monocytes [24], EC-derived EVs are involved in signaling pathways leading to altered VSMC phenotype [25], and EVs released by senescent VSMC modulate T cells and monocyte activity towards the proinflammatory phenotype [26]. On the other hand, although microcalcifications are known to promote macrophage proinflammatory responses, the role of calcifying EVs in the stimulation of inflammation is currently unknown. Though there is recognition of the diversity of EVs deposited on calcified tissues associated with different pathological conditions [7,20,27,28] as well as the importance of these tissue-trapped EVs [29], most studies have focused on the characterization of EVs released to 2D cell culture media, while those deposited in the ECM have remained largely understudied.

Gla-rich protein (GRP), also known as the upper zone of growth plate and cartilage matrix associated protein (UCMA) [30,31], has been shown to participate in multiple processes associated with VC at systemic and tissue levels [8,32,33]. It acts as a calcification inhibitor by reducing VSMC osteochondrogenic differentiation, at least in part through bone morphogenetic protein 2 (BMP2) binding and by decreasing mineral growth and maturation [32,33]. Lower GRP levels were associated with the calcification potential of VSMC-derived EVs, while increased levels decreased the pro-inflammatory reactions of multiple cells, including VSMCs and macrophages [10,32,34]. However, GRP has been shown to promote osteoblast differentiation and calcification [35,36], suggesting different modes of action in vascular and bone tissues.

In this work, we aimed to further explore the GRP molecular mechanism in VC associated with VSMC osteochondrogenic differentiation and calcifying of EVs, using add-of-function assays in ex vivo aortic fragments and human primary VSMC calcification models. Also, considering the heterogeneity of EVs known to be associated with calcified vascular tissues and the insufficient knowledge regarding EVs association with VC, particularly those trapped in the ECM of calcified tissues, we simultaneously characterized VSMC EVs released to the cell culture media and deposited in the ECM. Considering the interplay between VC and inflammation, and the role of EVs in intercellular communication, we evaluated a possible role of calcifying EVs in modulating macrophage inflammation. Overall, our data highlight the complexity of EVs released by VSMCs, with different populations carrying unique protein cargo, targeted to specific locations and distinct capacities to promote macrophage inflammation. Also, we reinforce the function of GRP as an inhibitor of VSMC osteogenic differentiation and as an important factor in the calcification potential of EVs, acting as a potential link in the immunomodulatory capacity of calcifying EVs.

## 2. Results

### 2.1. Vascular Calcification Inhibitory Effect of GRP Is Mediated by Downregulation of Osteogenic-Related Genes and Upregulation of Mineralization Inhibitors

**Figure 1:**
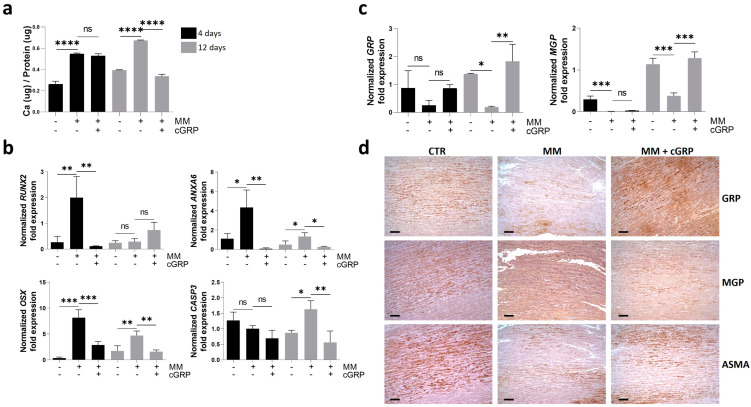
γ-carboxylated GRP (cGRP) inhibits VSMC osteogenic differentiation in ex vivo aortic fragments by downregulation of osteogenic-related genes and upregulation of mineralization inhibitors. Aortic fragments were cultured under control, mineralization (MM), and MM supplemented with cGRP media conditions for 4 (black bars) and/or 12 days (grey bars). (**a**) Calcium (Ca) quantification normalized to total protein levels. (**b**) Relative gene expression by qPCR of the osteogenic markers runt-related transcription factor 2 (*RUNX2*), osterix (*OSX*), annexin A6 (*ANXA6*), and the apoptotic marker caspase 3 (*CASP3*). (**c**) Relative gene expression by qPCR of the mineralization inhibitors gla rich protein (*GRP*) and matrix gla protein (*MGP*). SD was calculated from 3 independent experiments (*n* = 3) and ANOVA, with comparison between groups by the Dunnett test, was performed relative to the MM condition. Statistical significance was defined as *p* ≤ 0.05 (*), *p* ≤ 0.01 (**), *p* ≤ 0.001 (***), and *p* ≤ 0.0001 (****); ns, non-significant. (**d**) Representative immunohistochemical (IHC) experiments in consecutive tissue sections of aortic fragments cultured for 12 days in control (CTR), MM, and MM supplemented with cGRP (MM + cGRP) conditions, to detect GRP, MGP, and α-smooth muscle actin (ASMA). Gamma adjustments were performed to homogenize the panel and were applied to the entire image. Scale bar, 100 μm.

Considering that one of the major mechanisms mediating GRP’s VC inhibitory effect has been shown through the modulation of VSMC osteochondrogenic differentiation, we further characterized the cellular responses and molecular mechanisms involved in the calcification inhibitory effect mediated by gamma-carboxylated GRP (cGRP) over time using the ex vivo aortic fragments model [8]. The calcium (Ca) quantification and gene expression profile of known calcification-related gene markers were determined at days 4 and 12 to follow the mineralization process in aortic fragments, cultured with or without cGRP supplementation. Although an increase in Ca content was already evident at day 4 in mineralizing conditions (MM), decreased Ca levels resulting from cGRP supplementation were only detected after 12 days of treatment (Figure 1a). Gene expression analysis determined by qPCR of the osteogenic factors runt-related transcription factor 2 (*RUNX2*), osterix (*OSX*), and annexin A6 (*ANXA6*) showed a strong upregulation in MM conditions at day 4 (Figure 1b). The increased *OSX* and *ANXA6* expression was maintained at day 12 in MM conditions, although at lower levels than at day 4. Supplementation with cGRP rescued the upregulation of all three genes to control levels at day 4 as well as *OSX* and *ANXA6* expression at day 12 (Figure 1b). Caspase 3 (*CASP3*) gene expression was upregulated in MM conditions at day 12, indicative of apoptosis occurring in mineralizing VSMCs, while cGRP supplementation was able to retain *CASP3* expression at control levels (Figure 1b).

In addition, the gene expression analysis of *GRP* and *MGP* during the mineralization process showed a strong down-regulation of both genes at day 12 (Figure 1c). Supplementation with cGRP induced a self-upregulation and an upregulation of *MGP* at day 12 (Figure 1c). To further confirm the gene expression results, patterns of GRP and MGP accumulation were evaluated at day 12 by IHC of consecutive aortic tissue sections. In control artery tissues a strong signal for total GRP and total MGP was detected with similar patterns, indicating high levels of protein production (Figure 1d). A reduction of GRP, MGP, and α-smooth muscle actin (ASMA) was observed in tissues cultured in MM conditions, and supplementation with cGRP seemed to restore GRP, MGP and, to a lesser extent, ASMA production in VSMCs (Figure 1d). The positive intracellular signal detected by IHC and the upregulation of GRP gene expression indicate a positive feedback mechanism of GRP self-upregulation. Negative controls showed the absence of a signal (Figure S1).

Overall, these results indicate that GRP protects VSMC from calcification by the downregulation of genes known to be crucial factors in the onset of the osteochondrogenic differentiation process while increasing levels of calcification inhibitors, to maintain VSMCs in a contractile phenotype.

### 2.2. Calcification of Aortic Tissues Is Associated with Extracellular Vesicles (EVs)

**Figure 2:**
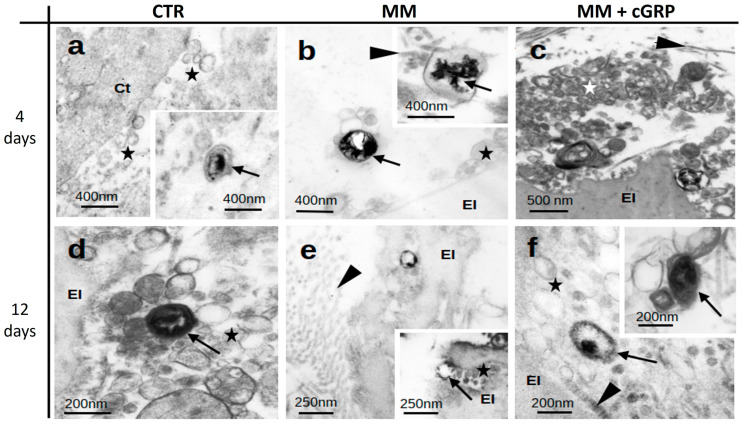
Aortic tissue calcification is associated with calcified extracellular vesicles (EVs). Representative images of transmission electron microscopy (TEM) analysis of aortic tissues of Epon-Araldite resin ultrathin sections, cultured under control (CTR) (**a**,**d**), mineralizing (MM) (**b**,**e**), and MM supplemented with cGRP (MM + cGRP) (**c**,**f**) media for 4 and 12 days. Stars indicate extracellular vesicles; arrows indicate mineralized vesicles; arrowheads indicate collagen; El, elastin; Ct, cytoplasm of smooth muscle cell.

Ultrastructural characterization of aortic tissues by transmission electron microscopy (TEM) revealed the presence of numerous EVs in all tissues analyzed from control (CTR), mineralizing (MM), and MM supplemented with cGRP (MM + cGRP) culture conditions (Figure 2). The EVs were found to be highly heterogeneous, differing in size (ranging from 50 to 200 nm approximately), density, and morphology. In CTR tissues, electron-dense mineralized EVs were occasionally detected at days 4 and 12 (Figure 2a,d), most probably reflecting previously existent tissue calcification. In MM conditions, the presence of EVs containing needle-like inclusions compatible with calcium phosphate mineral structures were detected both in the lumen and membrane of EVs (Figure 2b,e). In MM conditions supplemented with cGRP, large clusters of EVs were observed after 4 days. Some of these displayed dense cores, suggestive of non-crystalline (amorphous) calcium phosphate mineral (Figure 2c,f). EVs were detected in association with smooth muscle fibers, elastin, and collagen fibrils in all tissues (Figure 2b–f). EVs were clearly present near the surface of elastin bodies (Figure 2b–f) or embedded within channels of the elastin fibers (Figure 2e inset) but not extending into these extracellular matrix (ECM) components.

### 2.3. VSMCs Release Complex and Heterogeneous Populations of Extracellular Vesicles

Considering the importance of EVs as mediators of vascular calcification and the lack of a complete characterization of these heterogeneous EV populations, particularly for ECM-calcifying EVs, we further focused on the characterization of media-released and ECM-deposited EVs using the widely accepted human primary VSMC calcification model.

**Figure 3:**
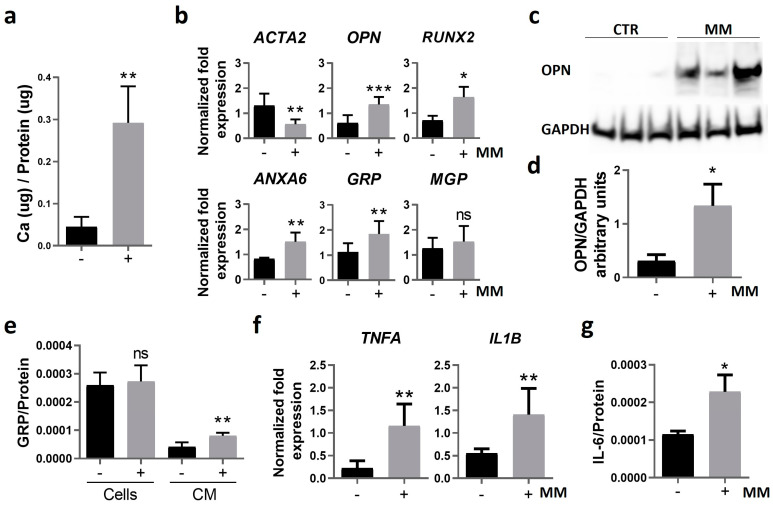
Calcification of human primary VSMCs is characterized by osteoblastic differentiation and increased inflammation. Human primary VSMCs were cultured in control and mineralizing (MM) conditions for 14 days. (**a**) The mineralization rate was determined through calcium quantification normalized to protein levels. (**b**) Relative gene expression of VSMC osteogenic differentiation (*ACTA2* encoding for ASMA, osteopontin (*OPN*), *RUNX2*, *ANXA6*) and mineralization inhibitor (*GRP*, *MGP*) markers by qPCR. (**c**) Western blot analysis for OPN detection and glyceraldehyde 3-phosphate dehydrogenase (GAPDH) as loading control. Original gels are presented in Figure S2; (**d**) relative OPN protein levels normalized to GAPDH using ImageJ software version V1.53f51 (arbitrary units). (**e**) Quantification of GRP normalized to total protein levels in cell lysates (cells) and in the cell culture media (CM) by ELISA. (**f**) Relative gene expression of VSMC proinflammatory cytokines (tumor necrosis factor alpha, *TNFA*; interleukin-1 beta, *IL1B*), and (**g**) quantification of interleukin-6 (IL-6) released to the cell media by ELISA, normalized to total protein levels. In all experiments, SD was calculated from 3 independent experiments (*n* = 3), and unpaired *t* tests were used. Statistical significance was defined as *p* ≤ 0.05 (*), *p* ≤ 0.01 (**), *p* ≤ 0.001 (***); ns, non-significant. In all graphs, black bars correspond to analysis at 4 days, and grey bars to 12 days.

First, we characterized our system to confirm the effective induction of VSMC osteogenic differentiation and calcification, after 14 days in control (CTR) and mineralizing (MM) conditions. Increased Ca levels in MM VSMCs (Figure 3a) were accompanied by decreased gene expression of *ACTA2*, increased expression of osteopontin (*OPN*), *RUNX2*, *ANXA6,* and *GRP*, and no significant changes in *MGP* levels (Figure 3b). Increased OPN protein levels in MM VSMCs were also confirmed by Western blot analysis (Figure 3c,d). Intracellular and extracellular GRP levels were determined in VSMC protein extracts (Cells) and conditioned media (CM) by ELISA. The results showed that although intracellular GRP levels remained similar, GRP levels released to the CM were increased in MM VSMCs (Figure 3e), consistent with the upregulation of *GRP* gene expression.

In addition, since VC is known to be intimately correlated with inflammation [15,16], but with many open questions related to this interplay, we explored the effect of calcification on VSMC inflammatory status. Increased levels of *TNFA* and *IL1B* gene expression (Figure 3f), as well as increased accumulation of IL-6 in the cell culture media (Figure 3g) of MM VSMCs, indicate that calcification induces a pro-inflammatory response in VSMCs.

**Figure 4:**
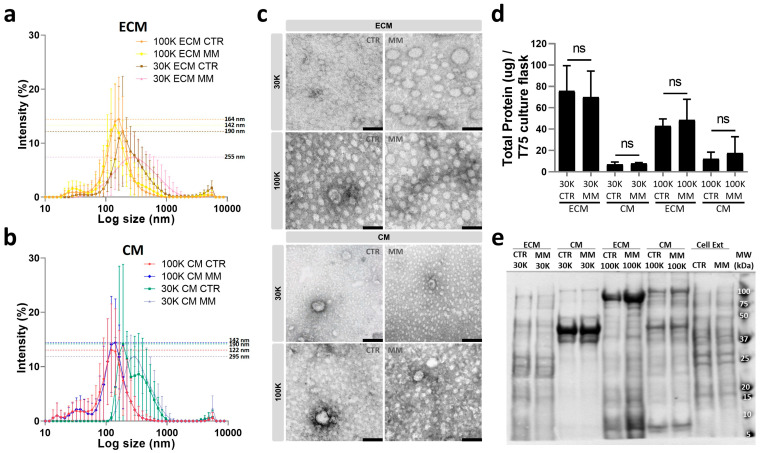
VSMC release different extracellular vesicle (EV) populations according to isolation centrifugation force and target location. EVs released to the cell media (CM) and deposited in the extracellular matrix (ECM) by human primary VSMCs cultured in control (CTR) and mineralizing (MM) conditions were isolated by differential ultracentrifugation at 30,000× *g* (30K) and 100,000× *g* (100K). (**a**,**b**) Particle size distribution by dynamic light scattering of EVs isolated at 100K and 30K from the ECM of CTR and MM VSMC (**a**), and from the CM of CTR and MM VSMC (**b**). Data are presented with SD from 3 independent isolation experiments (*n* = 3); non-significant differences were found between CTR and MM EVs within each population by unpaired *t* tests. (**c**) Representative images of transmission electron microscopy (TEM) analysis of all isolated EV populations. Scale bar, 100 nm. (**d**) Total protein quantification of isolated EV populations normalized to T75 confluent VSMC culture flask. SD was calculated from 3 independent experiments, and unpaired *t* tests were used: ns, non-significant. (**e**) Protein profile analyzed by SDS-PAGE of all isolated EV populations and VSMC protein extracts (Cell Ext). The positions of relevant molecular mass markers (kDa) are indicated on the right side.

EVs released to the cell culture media (CM EVs) and deposited in the ECM (ECM EVs) were isolated from CTR and MM VSMCs by sequential ultracentrifugation at 30,000× *g* (30K) and 100,000× *g* (100K), attempting to separate larger and smaller EVs and diminish the heterogeneity of each isolated population. Measurements of the particle size of isolated EV populations, determined by dynamic light scattering, showed an overall distribution profile more similar between centrifugation forces than sites of isolation, but mainly with a unimodal distribution in all populations (Figure 4a,b and Figure S3). However, 30K EVs showed a broader size distribution in relation to 100K EVs, indicative of bigger and more heterogeneous populations. Comparison between MM and CTR EVs showed no significant differences within each population. CTR and MM 100K EVs revealed highly similar size distribution, with a peak particle size of 164 nm for ECM CTR and 142 nm for ECM MM as well as 122 nm for CM CTR and 142 nm for CM MM. Although not significant, 30K CTR and MM EVs were found to be slightly more different, with a peak particle size of 190 nm for ECM CTR and 255 nm for ECM MM as well as 190 nm for CM CTR and 295 nm for CM MM. TEM analysis showed the presence of small intact vesicles in all populations, more variable in size on the 30K EVs isolated from all conditions, and more homogeneous populations in the 100K EVs, mostly smaller than 100 nm (Figure 4c). Total protein quantification showed higher protein levels in EVs isolated from the ECM when compared to the CM, for both 30K and 100K EVs, and similar levels between CTR and MM EVs in all populations (Figure 4d). SDS-PAGE analysis revealed a specific banding pattern for each population, different from cell extracts but similar between CTR and MM EVs for all populations (Figure 4e), suggesting substantial differences in the molecular composition of the EVs isolated at 30K and 100K from the ECM and CM.

Overall, these results suggest some overlapping physical characteristics but distinct biochemical features for the different isolated populations, highlighting the complexity and heterogeneity of EVs released by VSMCs.

### 2.4. Proteomic Characterization of VSMC EVs Identifies Different Populations with Specific Molecular Features and Regulated Proteins Associated with Vascular Calcification

**Figure 5:**
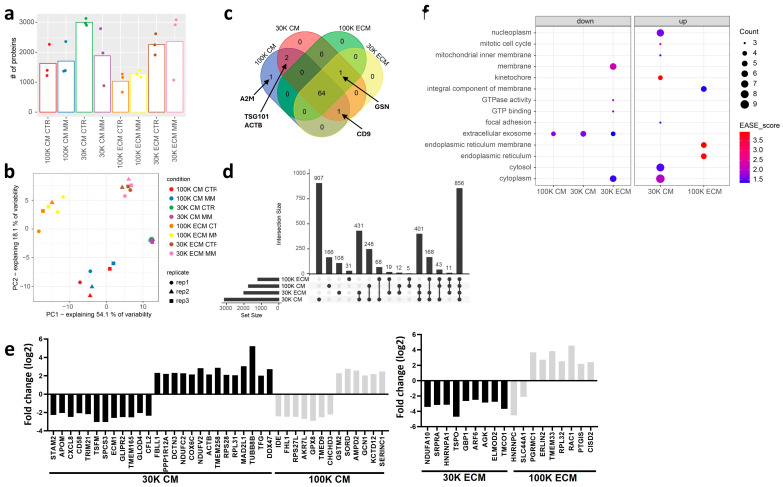
Proteomic characterization of VSMC EVs. (**a**) Protein identification rates per experimental condition (i.e., each of the eight combinations of EV subpopulation and culture condition). (**b**) Principal component analysis scatter plot, showing sample projections on the first two principle components. (**c**) Venn diagram relating the four EV populations in terms of the fraction of their protein content belonging to Vesiclepedia’s top 100 most frequently detected proteins. (**d**) UpSet diagram for the full protein sets detected in the four EV populations. Each column on the *x*-axis refers to a subset of proteins from the universe of 3774 present in a defined combination of the four EV populations (black dots). (**e**) Log2 fold changes of proteins whose levels are altered (adj. *p*-value < 0.05 and |log2 fold change| >2) in MM relative to CTR condition in each of the four sample types. (**f**) Bubble plot showing enriched GO terms (EASE SCORE < 0.05) in the lists of upregulated and downregulated proteins from each sample type’s MM to CTR comparison. STAM2, signal transducing adapter molecule 2; APOM, apolipoprotein M; CXCL8, interleukin-8; CD58, lymphocyte function-associated antigen 3; TRIM21, E3 ubiquitin-protein ligase TRIM21; TSFM, elongation factor Ts, mitochondrial; SPCS3, signal peptidase complex subunit 3; ECM1, extracellular matrix protein 1; GLIPR2, golgi-associated plant pathogenesis-related protein 1; TMEM165, transmembrane protein 165; GLOD4, glyoxalase domain-containing protein 4; CFL2, cofilin-2; FBLL1, rRNA/tRNA 2-O-methyltransferase fibrillarin-like protein 1; PPP1R12A, protein phosphatase 1 regulatory subunit 12A; DCTN3, dynactin subunit 3; NDUFC2, NADH dehydrogenase [ubiquinone] 1 subunit C2; COX6C, cytochrome c oxidase subunit 6C; NDUFV2, NADH dehydrogenase [ubiquinone] flavoprotein 2, mitochondrial; ACTB, actin, cytoplasmic 1; TMEM258, transmembrane protein 258; RPS28, 40S ribosomal protein S28; RPL31, 60S ribosomal protein L31; MAD2L1, mitotic spindle assembly checkpoint protein MAD2A; TUBB8B, tubulin beta-8 chain; TFG, protein TFG; DDX47, probable ATP-dependent RNA helicase DDX47; IDE, insulin-degrading enzyme; FHL1, four-and-a-half LIM domains protein 1; RPS27L, 40S ribosomal protein S27-like; AKR7L, aflatoxin B1 aldehyde reductase member 4; GPX8, glutathione peroxidase 8; TMED9, transmembrane emp24 domain-containing protein 9; CHCHD3, MICOS complex subunit MIC19; GSTM2, glutathione S-transferase Mu 2; SORD, sorbitol dehydrogenase; AMPD2, AMP deaminase 2; GCN1, eIF-2-alpha kinase activator GCN1; KCTD12, BTB/POZ domain-containing protein KCTD12; SERINC1, serine incorporator 1; NDUFA10, NADH dehydrogenase [ubiquinone] 1 alpha subcomplex subunit 10, mitochondrial; SRPRA, signal recognition particle receptor subunit alpha; HNRNPA1, heterogeneous nuclear ribonucleoprotein A1; TSPO, translocator protein; GBP1, guanylate-binding protein 1; ARF6, ADP-ribosylation factor 6; AGK, acylglycerol kinase, mitochondrial; ELMOD2, ELMO domain-containing protein 2; TMCO1, calcium load-activated calcium channel; HNRNPC, heterogeneous nuclear ribonucleoproteins C1/C2; SLC44A1, choline transporter-like protein 1; PGRMC1, membrane-associated progesterone receptor component 1; ERLIN2, erlin-2; TMEM33, transmembrane protein 33; RPL32, 60S ribosomal protein L32; RAC1, ras-related C3 botulinum toxin substrate 1; PTGIS, prostacyclin synthase; CISD2, CDGSH iron-sulfur domain-containing protein 2.

Proteome characterization of EV populations released by VSMC under CTR and MM conditions allowed the identification of 3.995 proteins (Table S1.1). Among these, the 30K EVs contained, in general, a higher number of proteins, particularly the 30K ECM, which showed similar numbers under both conditions, while the 30K CM under CTR had more proteins than the 30K CM under MM, (Figure 5a). The 100K EVs contained, in general, fewer proteins, with no major differences being found between CTR and MM conditions (Figure 5a). Principal component analysis performed on a list of 2382 proteins that were reliably quantified (Table S1.2, see Methods Section) clearly demonstrates different proteomes for each of the four isolated EV populations: 30K CM, 30K ECM, 100K CM, and 100K ECM, without clear differences between CTR and MM conditions (Figure 5b). Considering these similarities, protein groups from CTR and MM EVs were grouped for each population, 30K CM, 30K ECM, 100K CM, and 100K ECM, and the protein composition was further tightened through filtering parameters, considering only proteins found in at least half of the samples in each group. To investigate the extracellular vesicle nature of our samples, we started by performing a comparison with known EV markers from the top 100 Vesiclepedia protein list. A total of 69 top 100 Vesiclepedia proteins were identified. From these, 64 were common to all EV populations, including the most widely used EV markers, such as CD81 antigen (CD81), CD63 antigen (CD63), syntenin-1 (SDCBP), programmed cell death 6-interacting protein (PDCD6IP), and flotillin-1 (FLOT1), while CD9 antigen (CD9) was absent from 100K ECM EVs, gelsolin (GSN) was absent from 100K CM, tumor susceptibility gene 101 protein (TSG101), and actin, cytoplasmic 1 (ACTB) was only identified in CM EVs, both 100K and 30K, and alpha-2-macroglobulin (A2M) was exclusively present in 100K CM EVs (Figure 5c, Table S1.1). In addition, following MISEV 2018 guidelines and a bibliography search on recently suggested EV markers [18,19,37], we constructed different protein lists with markers known to be enriched in all EVs, small EVs (sEVs), medium/large EVs (m/l EVs), and bona fide exosomes (Table S1.3). Gene set enrichment analysis (GSEA) for all EVs showed similar enrichment scores (ES) of 0.6 for 100K CM and 100K ECM, while a slightly lower ES of 0.5 for 30K CM and ECM populations (Figure S3). All populations contained representatives of most protein categories suggested by MISEV 2018 as general markers of all EVs (Table S1.4). Considering a possible categorization into sEVs and m/l EVs, 100K and 30K CM EVs were found to be more enriched in sEV and m/l EV markers, respectively, as expected from the differential ultracentrifugation protocol and in accordance with the size distribution from DLS (Figure 4a). However, ECM EVs, both 100K and 30K, are not clearly enriched in sEVs or m/l EVs markers, suggesting a more complex and heterogeneous nature of ECM-derived EVs (Figure S3). Interestingly, when we consider markers suggested to be present exclusively in exosomes, the bona fide exosome markers [37], 100K CM EVs have the highest ES of 0.79, but 100K ECM EVs also have an ES score of 0.66. Albumin and IgGs were not identified in our data set, suggesting a low level of contamination from FBS.

Further characterization of the molecular profile of each population revealed 856 proteins common to all EV groups, while each group also presented a set of unique proteins, with 907 specific proteins in 30K CM, 108 in 30K ECM, 166 in 100K CM, and 31 in 100K ECM (Figure 5d, Table S1.1). To obtain functional insight into the common and unique EV proteomic cargos, we performed gene ontology (GO) analysis (Figure S4). The group of proteins common to all EV populations was found to be enriched in protein and RNA binding activities, associated with multiple cellular components, particularly cytosol, cytoplasm, and membrane and extracellular exosome, and involved in signal transduction. From the analysis of the unique proteins present in each population, it is interesting to note that the 100K ECM EV proteome stands out for its enrichment in RNA as well as its transcription- and translation-associated proteins at molecular function and biological processes levels; it is also associated with the nucleoplasm, nucleolus, and chromosome as the main cellular components. In contrast, 100K CM EVs’ unique proteins were highly enriched in protein and ion binding activity, involved in signal transduction, cell adhesion, and innate immune response, and highly associated with the extracellular exosome, cytosol, and the extracellular region and space. The unique 30K ECM proteome was found to be heavily overlapping with that of 100K CM EVs in terms of molecular function and biological processes but highly associated with plasma membrane components, mitochondria, and the endoplasmic reticulum membrane. Interestingly, although the 30K CM unique EV proteome contained the highest number of individual proteins (907), these were characterized by the lowest number of GO terms, including a combination of protein and RNA binding activities, involved in signal transduction and protein transport, localized at the cytosol, nucleus, and nucleoplasm, as well as membrane components including mitochondria. Overall, these data indicate that all populations were enriched in extracellular vesicles, but each presents unique molecular features, with specific functional protein groups in ECM and CM EVs, suggesting different cell origins. While specific characteristics of CM EVs are suggestive of a classical classification into small EVs enriched in exosomes in the 100K and medium/large EVs in the 30K, additional studies are required to elucidate ECM EVs’ nature.

Analysis of differentially expressed proteins between MM and CTR conditions showed the most pronounced differences among the CM EVs, particularly the 30K, with 12 downregulated and 14 upregulated proteins in MM. In CM 100K EVs, seven proteins were found downregulated and six upregulated in MM; in ECM 30K, nine proteins were downregulated and none upregulated in MM; and in ECM 100K, two were downregulated and seven were upregulated in MM (Figure 5e). The highest number of GO terms were found in the sets of proteins upregulated in MM conditions associated with the CM 30K and ECM 100K. In CM 30K, a higher number of core genes were associated with nucleoplasm, cytosol, and cytoplasm, with the highest EASE scores associated with the kinetochore and mitotic cell cycle, suggesting specific targeting of differentially regulated proteins associated with altered cell division during the mineralization process for this particular EV population (Figure 5f, Table S1.5). In ECM 100K, the highest EASE scores were associated with the endoplasmic reticulum membrane and endoplasmic reticulum, indicating that this EV population might be particularly involved in endoplasmic reticulum stress during the mineralization process (Figure 5f). When looking at enriched annotations amongst proteins downregulated in MM conditions, extracellular exosome is the common term, suggesting that CM 100K, CM 30K and ECM 30K populations are all involved in EV-mediated vascular calcification (Figure 5f). Indeed, by comparing our list of differentially regulated proteins with the specific EV proteomes of carotid atherosclerotic plaque and calcific aortic valve stenotic tissues recently published [29], 14 were identified in common with atherosclerosis progression, while only 7 of these 14 were common in calcific aortic valve disease progression (Table S1.6), supporting our VSMC mineralization model.

Next, we analyzed the specific reported functions of major regulated proteins in MM conditions. Identified proteins with the highest fold change have been associated with cell growth, proliferation, differentiation, migration, and ECM organization, such as tubulin beta-8 chain (TUBB8B), mitotic spindle assembly checkpoint protein MAD2A (MAD2L1), translocator protein (TSPO), heterogeneous nuclear ribonucleoproteins C1/C2 (HNRNPC), membrane-associated progesterone receptor component 1 (PGRMC1), and ADP-ribosylation factor 6 (ARF6); with protein translation, metabolism, and processing: elongation factor Ts (TSFM), signal peptidase complex subunit 3 (SPCS3), probable ATP-dependent RNA helicase DDX47 (DDX47); with regulation of calcium homeostasis and oxidative stress: glutathione peroxidase 8 (GPX8), aflatoxin B1 aldehyde reductase member 4 (AKR7L), NADH dehydrogenase [ubiquinone] flavoprotein 2 (NDUFV2), transmembrane protein 258 (TMEM258), glutathione S-transferase Mu 2 (GSTM2), sorbitol dehydrogenase (SORD), calcium load-activated calcium channel (TMCO1), and transmembrane protein 33 (TMEM33); inflammation and immune response: ras-related C3 botulinum toxin substrate 1 (RAC1), guanylate-binding protein 1 (GBP1), ELMO domain-containing protein 2 (ELMOD2), interleukin-8 (CXCL8), lymphocyte function-associated antigen 3 (CD58), and E3 ubiquitin-protein ligase TRIM21 (TRIM21); with the negative regulator of endochondral bone formation and mineralization: extracellular matrix protein 1 (ECM1). All these processes are widely known to be associated with vascular calcification [4,5,15].

Of special note, RAC1, which has been shown as an interesting link between inflammation and calcification in atherosclerosis, was found to be highly upregulated in 100K ECM MM [38].

### 2.5. ECM EVs from Mineralizing VSMCs with Higher Ca and Lower GRP Levels Induce a Low-Grade Inflammatory Response in Macrophages

**Figure 6:**
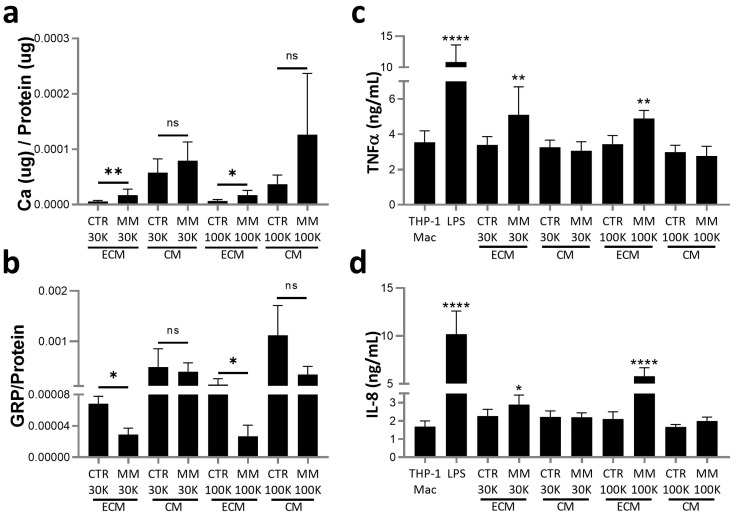
Extracellular matrix (ECM)-deposited EVs released by VSMCs under mineralization stress contain higher calcium (Ca) and decreased GRP levels and induce macrophage inflammation. (**a**) Calcium (Ca) content and (**b**) GRP quantification by ELISA, both normalized to protein levels, determined in VSMC-isolated EV populations as described in Figure 4 legend. SD was calculated from 3 independent experiments (*n* = 3), and unpaired *t* tests were used. Statistical significance was defined as *p* ≤ 0.05 (*), *p* ≤ 0.01 (**); ns, non-significant. (**c**,**d**) THP-1 differentiated macrophages (TPH-1 Mac) were treated with 5 ug/mL of VSMC EVs quantified according to total protein for each population, and the positive inflammatory control lipopolysaccharides (LPSs) (100 ng/mL), for 24 h. Levels of the proinflammatory cytokines TNFα (**c**) and interleukin-8 (IL-8) (**d**) released to the cell media were determined by ELISA. SD was calculated from 3 independent experiments (*n* = 3), and ANOVA, with comparison between groups by the Dunnett test, was performed relative to THP-1 Mac cells cultured in control conditions. Statistical significance was defined as *p* ≤ 0.05 (*), *p* ≤ 0.01 (**), and *p* ≤ 0.0001 (****).

Despite the different molecular cargo identified for each isolated population of EVs released by VSMCs, it is likely that several low abundant proteins associated with the molecular modulation of the mineralization process were not detected, or identified as differentially expressed, by our proteomic profiling approach. Given our particular focus on the association of GRP with calcifying EVs, we evaluated GRP levels and quantified calcium as indicators of calcifying potential. Although higher levels of Ca were found in CM EVs, in both 30K and 100K populations, only the ECM-EVs showed differences between CTR and MM EVs, with higher levels in both 30K ECM MM and 100K ECM MM relative to respective CTR EVs (Figure 6a). GRP levels were found to be inversely related to Ca, with lower GRP levels in 30K and 100K ECM MM EVs relative to the CTR EVs (Figure 6b). To evaluate a possible role of EVs released under calcification stress in mediating inflammatory responses, functional assays were performed in THP1-differentiated macrophages (THP1-Mac) treated for 24 h within each of the isolated populations and measuring the levels of pro-inflammatory cytokines. When compared to cells maintained in control conditions or treated with CTR EVs, THP1-Mac cells treated with 30K and 100K ECM MM-EVs released higher levels of both TNFα and IL-8, although never reaching the levels produced upon classical inflammation stimulation with LPS (Figure 6c,d). Overall, these results indicate that ECM EVs released under calcification stress with higher Ca and decreased GRP levels, suggestive of increased calcification potential, are able to induce a low-grade chronic inflammatory response in inflammatory cells such as tissue resident macrophages.

## 3. Discussion

In the present work, we show that gamma-carboxylated GRP (cGRP) is involved in multiple processes occurring in VC, particularly in the osteogenic differentiation of VSMCs, the calcifying potential of EVs, and immunomodulatory properties of calcifying EVs. We demonstrated that VSMCs release different EV populations with specific molecular features that seem to be targeted to the cell media (CM) or to the extracellular matrix (ECM). EVs released under calcification stress and deposited in the extracellular matrix, with increased calcium content, decreased GRP, and increased RAC1 protein levels, are able to modulate proinflammatory responses of macrophages. These data suggest that specific mineralization-competent EVs released by calcifying VSMCs have an active functional role in stimulating and maintaining a low-grade inflammatory response at microcalcification sites in the vasculature, which in turn contributes to perpetuate and propagate vascular calcification and inflammation.

### 3.1. The Role of GRP in VSMC Osteochondrogenic Differentiation

Using a previously validated model of vascular calcification with ex vivo human aortic fragments [8], we demonstrated that supplementation with cGRP induces a downregulation of the critical osteoblastic differentiation factors RUNX2 and OSX at early stages and an upregulation of the mineralization inhibitors MGP and GRP later on the mineralization process. These data are in clear agreement with the proposed role for GRP on the inhibition of osteo/chondrogenic differentiation of VSMC [8,33] and corroborate previous results showing increased expression of RUNX2 and decreased expression of MGP in VSMCs from GRP^−/−^ mice [33]. At a molecular level, this sequence of events can be explained by the proposed role of cGRP in inhibiting the BMP2 signaling pathway through direct BMP2 binding and a consequent decrease in the phosphorylation of SMAD1/5/8, with downstream effects on the cascade of osteogenic differentiation of VSMCs [33]. Our data on cGRP-treated aortic tissues showing decreased levels of RUNX2 and OSX earlier in the mineralization process and a prolonged effect on OSX in the latter stages is in concordance with the knowledge that BMP2-induced OSX is downstream of RUNX2 [39]. Although a potential role for RUNX2 in the regulation of GRP and MGP has been suggested, contradictory data indicate complex regulations of GRP and MGP according to specific tissues. In osteoblasts, GRP was shown to be upregulated through the direct transcription activation by RUNX2 and OSX, with a consequent increase in calcified nodule formation [35], while an opposite effect was shown in primary osteoblasts, mesenchymal stem cells, and MC3T3-E1 pre-osteoblasts, where GRP impaired osteogenesis [31]. Also, our present data showing decreased RUNX2 and OSX and increased GRP levels with cGRP treatments preclude a positive regulation of GRP by RUNX2, in agreement with previously reported results showing a calcification inhibitory function for GRP in vascular tissues [8,33]. Interestingly, similar conflicting data have also been reported for the transcriptional activation of MGP by RUNX2, associated with bone and vascular calcification. In osteoblasts, MGP was shown to increase osteoblast proliferation, differentiation, and mineralization through Wnt/β-catenin and RUNX2 signaling pathways [40], also in agreement with the MGP upregulation in osteoblastic cells overexpressing RUNX2 [41]. In vascular tissues MGP is an established calcification inhibitor with non-related RUNX2 expression patterns; RUNX2^−/−^ mice exhibit normal MGP expression, indicating that MGP is not under the direct control of RUNX2 [42,43].

Importantly, we show that the delayed transformation of VSMCs is accompanied by decreased calcification of the extracellular matrix, mainly associated with EVs. Ultrastructural analysis reveals that most calcified material is found within EVs, and treatments with cGRP results in the release of EVs to the ECM devoid of needle-like structures, suggesting decreased mineral crystallinity. This is consistent with previous data showing that cGRP inhibits mineral maturation and growth and that calcifying EVs from mineralized aortic tissues contain lower GRP levels [8,32]. In addition, our ultrastructural analysis shows the presence of calcification associated with the lumen and membrane of morphologically distinct EVs. This supports the notion that different EVs participate in the VC process, which initiates with the maturation of amorphous calcium phosphate into crystalline hydroxyapatite that radiates towards the lumen of EVs to the ECM, acting as a nidus for initial mineral deposition [6,44,45]. However, though ECM-associated EVs are known to have an active role in VC, the characterization of VSMC EVs has mainly focused on those released to cell culture media [25,27], leaving an important knowledge gap in the functional role of EVs deposited in the ECM in VC.

### 3.2. VCMC Release Different EV Populations Targeted to the Extracellular Matrix and to the Cell Media

Considering the challenges associated with the isolation of vascular tissue-associated EVs, we selected the widely accepted in vitro model of VC using primary human VSMCs induced to mineralize in high phosphate conditions, to simultaneously isolate and characterize EVs deposited in the ECM (ECM-EVs) and released to the cell culture media (CM-EVs). The use of this 2D in vitro model combined with the ECM digestion with collagenase circumvent the necessity of mechanical homogenization usually associated with tissue disruption [46]. This approach enables the digestion of the collagen-rich ECM characteristic of VSMCs, maintaining cell integrity and minimizing co-isolation of intracellular vesicles or membranous particles [29]. Also, the differential centrifugation approach using 30,000× *g* (30K) followed by 100,000× *g* (100K) allows the separation of larger and smaller vesicles, decreasing sample complexity and heterogeneity [18,19,37].

Overall, our physical, biochemical and proteomic characterization of isolated VSMC-EVs indicate that ECM- and CM-EVs, both 100K and 30K, have distinct molecular features but share a common footprint of EV markers. All populations were shown to contain representatives of most protein categories suggested by MISEV 2018 [19] as general markers of all EVs. These data, combined with TEM analysis and association of these common proteins to cytosol, cytoplasm, membrane, and extracellular exosomes, indicate that all sample populations are, at least in part, of extracellular vesicle origin. To further establish the specific features of each EV population, we used a combined strategy of gene ontology analysis for the specific protein groups assigned to each population and established enrichment scores with protein lists constructed based on recent studies focused on the establishment of protein markers for EVs subtypes, particularly small (sEVs) and medium/large EVs (m/lEVs) and bona fide exosomes [18,19,37].

Overall, our data suggest a classical categorization of 100K CM EVs as sEVs enriched in exosomes, and the 30K CM population enriched in medium/large EVs, in agreement with the differential centrifugation protocol, size distribution, and TEM characterization. Also, EVs isolated at 100,000× *g* from VSMC culture media were previously identified as exosomes [27].

However, more complex data were obtained for the characterization of ECM EVs, either reflecting more heterogenous populations containing both sEVs and m/lEVs, or still poorly characterized EVs subtypes. Both 100K and 30K ECM-EV populations are more highly enriched in sEV than m/lEV markers, despite the fact that these two populations were isolated through different centrifugation forces and present different size distributions, with 30K ECM containing slightly higher EV particle size. Also, the unique proteome of each population strongly suggests different subtypes from different cell origins. Specific proteins in 100K ECM EV were highly associated with the nucleus and RNA binding functionalities, while in 30K ECM EV, these were particularly associated with integral plasma membrane components, including organelles such as mitochondria and endoplasmic reticulum (ER). Mitochondria and ER proteins have been linked to m/lEVs, but both sEVs and m/lEVs are known to be enriched in plasma membrane proteins [18]. Also, different sEV subtypes can originate along different stages of the endosomal pathway [18,37], and definitive markers to identify EVs based on biogenesis are not yet established. The presence of nuclear and RNA-binding proteins (RBPs) has been mainly associated with sEVs [47], while others suggested its enrichment in m/lEVs from different breast cancer cell lines [18]. In fact, RBPs with functions in RNA metabolism regulation have been suggested as playing a significant role in the sorting of coding and non-coding RNA in sEVs and delivered to receipt cells, influencing their biology [47,48]. Although we cannot establish the biogenesis of the 100K ECM EV population, its enrichment in bona fide exosome markers such as ADAM10 [37] suggest that these might be enriched in sEVs.

Interestingly, in our dataset we found two members of the heterogeneous nuclear ribonucleoproteins (hnRNPs) family, the HNRNPA1 and HNRNPC, as differentially regulated in ECM CTR and MM EVs. HNRNPs are known to be involved in several RNA-associated processes, such as pre-mRNA processing, splicing, and transport, and HNRNPA1 and HNRNPC have been suggested to be involved in miRNA loading into sEVs [49,50]. Since miRNAs are known to regulate several key cellular processes involved in vascular calcification [51], we can suggest a potential interaction network inside EVs to regulate the specific miRNAs involved in the mineralization process, worth exploring in the future. In addition, several other proteins were found to be differentially regulated between CTR and MM conditions in all EV populations, although each population contains specific regulated protein sets. Globally, these proteins are involved in multiple processes known to be associated with vascular calcification, such as cell growth, proliferation, differentiation, and migration, ECM organization, regulation of calcium homeostasis and oxidative stress, inflammation and immune response, and mineralization regulation [4,5,15]. Considering that our mineralizing cell model system was shown to induce osteochondrogenic differentiation and inflammation on VSMCs, these data indicate that the cell environment under mineralization stress is, at least in part, reflected in the EV content. However, despite the relative high number of proteins identified in our VSMC EV populations when compared to other studies [25,27], the number of differentially regulated proteins between CTR and MM conditions was relatively low, particularly on the 100K EVs. Since calcifying EVs have been demonstrated as being isolated after short ultracentrifugation periods [52], a possible explanation might be related to our ultracentrifugation protocol performed in the same conditions for CTR and MM EVs, probably reducing the signal-to-noise ratio of specific calcification-related proteins. This might also explain the non-differentiated proteomes obtained for CTR and MM EVs in all populations by PCA analysis.

### 3.3. EVs as a New Signaling Axis Between Vascular Calcification and Inflammation

The effect of VSMC-derived EVs on the ECM calcification has been attributed to their calcification potential, highly associated with increased calcium content and decreased levels of calcification inhibitors [6,8,27]. Our data showing increased calcium and decreased GRP levels in ECM MM EVs, both in 100K and 30K, indicates that these EV populations have higher calcifying potential, reinforcing their active role in ECM mineralization. Importantly, we present the first evidence that these ECM MM EVs are able to functionally alter macrophage inflammatory status by inducing a significant increase in proinflammatory cytokine production. Interestingly, EVs isolated from the ECM of mouse calcifying VSMCs, contrarily to EVs isolated from the CM, were shown to induce vascular calcification in non-calcifying VSMCs and were suggested as a mechanism to propagate microcalcification throughout the vascular tissue [53]. Also, EVs released by senescent VSMCs were shown to modulate T cells and monocyte activity towards a proinflammatory phenotype [26]. This is in line with our data, demonstrating specific functionalities for the different EV populations released by VSMCs and the capacity of VSMC-released EVs in influencing inflammation. Although the mechanism involved in macrophage activation by ECM MM EVs is currently unknown, the higher calcium and decreased GRP levels are known to relate to macrophage proinflammatory states. Basic calcium phosphate crystals (BCPs) likely occurring in calcified EVs directly interact with macrophages, eliciting proinflammatory responses [9]. GRP has been demonstrated as an anti-inflammatory agent, inducing decreased production and accumulation of proinflammatory cytokines and inflammation mediators such as TNFα, IL-6, IL-1β, IL-8, cyclooxygenase 1 (COX2), nuclear factor kappa-light-chain-enhancer of activated B cells (NFKB), and prostaglandin E2 (PGE2) [10,32,54]. In a clinical context, decreased GRP serum levels were associated with increased IL-6, TNFα, and C-reactive protein levels in disease cohorts [55,56]. Also, RAC1, an important signal transducer of inflammatory cytokine expression, was found as the highest upregulated protein in 100K ECM MM EVs, providing an additional potential link for the functional proinflammatory effect of this population on macrophages. Indeed, Rac1 has been demonstrated to be a major regulatory determinant of macrophage IL-1β, which in turn is a key mechanism in promoting atherosclerosis calcification [14,38]. Our results suggest that in addition to the reported role of macrophage RAC1 in promoting calcification, VSMC RAC1 delivered through EVs might modulate macrophage inflammation.

## 4. Materials and Methods

### 4.1. Ethical Approval and Aortic Segment Ex Vivo Calcification Assays

Fragments of human ascending thoracic aorta were obtained from four patients, 2 females and 2 males (age 66 ± 10 years), diagnosed with aortic valve stenosis, at the time of aortic valve replacement at Service of Cardiothoracic Surgery, Santa Cruz Hospital, CHLO, Lisbon, Portugal. This study was approved by the hospital Ethics Committee (2024-112). All principles of the Declaration of Helsinki were followed, and study procedures were only conducted after obtaining patients’ written informed consent. Aortic segment calcification assays were performed as previously described [8], cultured either in control conditions (CTR: DMEM/10% FBS/1% PS/1% L-Gln), in mineralizing conditions with high phosphate (P) and calcium (Ca) (MM: CTR supplemented with 3.6 mM Ca and 1.6 mM P), or in MM supplemented with 500 ng/mL of sturgeon GRP, further referred to as cGRP, for 12 days. At the end of the experiments, aortic segments were washed 3 times in PBS and immediately processed for Ca and protein quantification, gene expression, and immunohistochemistry (IHC) and TEM analysis, as described below.

### 4.2. VSMC Calcification Assays

Human aortic VSMCs derived from tissue explants were kindly provided by Prof. Dr. Leon Schurgers, Department of Biochemistry, CARIM, Maastricht University, The Netherlands, and used in calcification assays as previously described, always using EV-depleted FBS [32]. VSMCs were either cultured in control (CTR: M199/10% FBS/1% PS) or mineralizing (MM) conditions by media supplementation with 2.5 mmol/L NaH_2_PO_4_ for 13 days, with the media changed every 3 days, and then supplementation with 3.6 mmol/L CaCl_2_ for 24 h. For EV isolation, at least 2 confluent T75 flasks were used per condition, and molecular characterization experiments were performed in parallel in 6-well plates. At least three independent experiments were performed for EV isolation and characterization, and three independent experiments, each with triplicates per condition, were conducted for VSMC molecular characterization. At the end of the experiments, cell culture media (CM) was collected, and cells were washed 2 times with PBS before further experiments.

### 4.3. Extracellular Vesicle (EV) Isolation

EVs were isolated from CTR and MM VSMC culture media (CM) and the extracellular matrix (ECM) by differential ultracentrifugation at 30,000× *g* (30K) and 100,000× *g* (100K) by adapting previously described procedures [53]. EVs were released from the ECM by enzymatic digestion with 500 units/mL of collagenase (Gibco) in a buffer containing 0.25 M sucrose, 0.12 M NaCl, 0.01 M KCl, and 0.02 M Tris buffer pH 7.5, for 3 h at 37 °C with gentle agitation. The digestion mix was centrifuged at 2500 rpm for 30 min at 4 °C, and the recovered supernatant was centrifuged at 30,000× *g* for 30 min to obtain the 30K EVs, while the remaining supernatant was centrifuged again at 100,000× *g* for 2 h to obtain the 100K EVs. For isolation of the CM EVs, the same procedure was followed, starting with the removal of cell debris by centrifugation at 2500 rpm for 30 min at 4 °C. Centrifugations at 30,000 and 100,000× *g* were performed in a Beckman Otima XPN100 ultracentrifuge using the T70 fixed angel rotor. All EV pellets at 30K and 100K were washed with PBS and re-centrifuged in the respective conditions in a Beckman Otima Max XP micro-ultracentrifuge using the TLA-55 fixed angel rotor. All EV pellets were resuspended in PBS and immediately used for size distribution analysis; pellets were stored at −80 °C until further use [57].

### 4.4. Calcium and Total Protein Quantification

Calcium (Ca) quantification was performed in nitric acid digested aortic tissue fragments and in 1M HCl demineralized VSMCs by O-cresolphthalein complexone chemistry using a colorimetric assay (Randox Laboratories, Northern Ireland, UK) according to manufacturer’s recommendations. EVs resuspended in PBS were digested with nitric acid, and calcium was quantified by microwave plasma atomic emission spectroscopy (MP-AES) through a 4200 MP-AES (Agilent Technologies, Mulgrave, Victoria, Australia). Total protein extracts from aortic fragments were obtained with radioimmunoprecipitation assay (RIPA) buffer, as described [8]. VSMC protein extracts were obtained after demineralization with 1M HCl using 1M NaOH with 5% (*w*/*v*) SDS for neutralization and extraction. Isolated EV total protein quantity was directly obtained from intact EVs resuspended in PBS and is presented as EV total protein per T75 flask. In all cases, total protein quantification was determined by the MicroBCA protein assay kit (Pierce, Thermo Fisher Scientific, Waltham, MA, USA), and calcium results are presented normalized to protein quantity as calcium/protein ratios.

### 4.5. RNA Extraction and Gene Expression

Total RNA was extracted from aortic fragment tissues as described by Chomczynski and Sacchi [58], and from VSMCs using Direct-zol RNA Miniprep kit (Zymo Research, Irvine, CA, USA), according to the manufacturer’s instructions. Gene expression analysis was performed as previously described [8,32] with specific primer sets (Table S2) and glyceraldehyde-3-phosphate dehydrogenase (GAPDH) as the housekeeping gene.

### 4.6. Immunohistochemistry

Aortic fragment tissues collected into 4% (*w*/*v*) PFA solution and paraffin-embedded were used to detect total GRP and matrix gla protein (MGP) and actin alpha 2 (ASMA), using the CTerm-GRP (5 ug/mL, GenoGla Diagnostics, Faro, Portugal), MGP (1:1000, VitaK BV, Maastricht, The Netherlands), and ASMA (1:100, Santa Cruz Biotechnology, Dallas, TX, USA) primary antibodies, as described [8], and counterstained with hematoxylin-eosin. Negative controls consisted of omitting the primary antibody. Microphotographs were acquired with a Zeiss AXIOIMAGER Z2 microscope, with an AxioCam ICc3 camera and AxioVision software version 4.8 (Carl Zeiss, Baden-Württemberg, Germany).

### 4.7. Transmission Electron Microscopy (TEM)

Aortic tissue fragments were fixed in a solution containing 3% glutaraldehyde in 0.1 M sodium cacodylate buffer pH 7.4 for 3 h. After rinsing with cacodylate buffer, tissues were post-fixed with a 1% osmium tetroxide solution in 0.1 mol/L cacodylate buffer for 1 h at room temperature. Cells were then fixed with 0.5% uranyl acetate in citrate-acetic acid buffer pH 5.6 and dehydrated by gradually increasing ethanol concentration (70%, 95%, and 100% in bi-distilled water). The tissues were embedded in an Epon-Araldite mixture. Ultrathin sections were observed and photographed with a JEOL 1200EX transmission electron microscope after double staining with 2% uranyl acetate and lead citrate. EVs isolated at 30K and 100K from the ECM and CM were adsorbed onto formvar-carbon coated grids and stained with 1% aqueous uranyl acetate. The grids were air-dried, observed, and photographed using a JEOL 1200EX transmission electron microscope.

### 4.8. Electrophoresis and Western Blot

Aliquots of protein were size-separated on 4–12% (*w*/*v*) gradient polyacrylamide precast gels containing 0.1% (*w*/*v*) SDS (NuPage, Invitrogen, Thermo Fisher Scientific, Waltham, MA, USA) and either stained with G-250 Coomassie brilliant blue or transferred onto a nitrocellulose membrane (BioRad, Hercules, CA, USA) as previously described [8]. Osteopontin (OPN) and GAPDH were immunodetected through overnight incubation with OPN and GAPDH primary antibodies (1:100 and 1:500, respectively, Santa Cruz Biotechnology), species-specific secondary horseradish peroxidase-conjugated antibodies (Sigma, Livonia, MI, USA), and Western Lightning Plus-ECL (PerkinElmer, Hopkinton, MA, USA). Images were acquired in an IQ LAS 4000 mini biomolecular imager (GE Healthcare, Chicago, IL, USA). Quantification of protein levels was performed by measuring optical densities (ODs) using ImageJ software (version V1.53f51).

### 4.9. ELISA Assays

Levels of TNFα, interleukin-6 (IL-6), and IL-8 were determined in the CM of VSMCs and THP1 differentiated macrophages (THP-1Mac) using specific ELISA assays from R&D Systems. Extracellular and intracellular GRP levels were quantified in the CM and total protein extracts from VSMCs, and in RIPA extracts of isolated EVs, using a specific and validated ELISA assay for total GRP [32]. GRP levels were always normalized to respective total protein content and are presented as GRP/total protein ratios.

### 4.10. Extracellular Vesicle Size Distribution

Isolated EVs were analyzed by dynamic light scattering with a Zetasizer Nano ZS (Malvern Instruments, Westborough, MA, USA) immediately after isolation and resuspension in PBS by diluting each 10 μL of EV sample with 1 mL of milliQ water.

### 4.11. Proteomic Analysis

Proteins were extracted from EV samples using an Amphipol A8–35 polymer protocol, followed by a step of reduction and alkylation of cysteines. Digestion into peptides was carried out with lysyl endopeptidase and trypsin, and a final purification step cleared peptide mixtures of superfluous reagents. Samples were dried and stored at −20 °C.

For LC-MS/MS analysis, peptides were re-dissolved in loading solvent A (0.1% TFA in water/acetonitrile (ACN) (98:2, *v*/*v*)) and injected on an Ultimate 3000 RSLCnano system in-line connected to a Q Exactive HF mass spectrometer (Thermo Fisher Scientific, Waltham, MA, USA). The peptides were separated on a 250 mm analytical column, using a 150 min non-linear reverse-phase buffer gradient. The mass spectrometer was operated in data-dependent mode, with full-scan MS spectra (375–1500 *m*/*z*) acquisition at a resolution of 60,000 in the Orbitrap analyzer, followed by isolation of up to the 12 most intense ions for MS/MS also in the Orbitrap analyzer, after fragmentation by high-energy collisional dissociation fragmentation.

For protein identification and quantification, RAW Thermo files from triplicates of the eight different EV particle samples were searched using MaxQuant (version 2.0.3.0) against the human Uniprot/SwissProt database, leading to the identification of 3995 proteins. Protein label-free quantification was carried out by the MaxLFQ algorithm and by the iBAQ calculation tool integrated in the MaxQuant software. Further data analysis of the shotgun results was mainly performed in R programming language, using in-house scripts. Characterization of the four EV-particle sample types, 30K CM, 30K ECM, 100K CM, and 100K ECM, was performed on the list of 3474 proteins detected in three or more samples out of the six representatives of each group. Functional enrichment analysis was performed using DAVID [59] on the list of proteins common to all EV-particle purification sample types, as well as on the four lists of proteins exclusive to each of these four sample types. Gene set enrichment analysis [60] was performed for the four EV-particle purification sample types, using as metric lists of median protein relative abundance and as gene sets four distinct groups of EV markers [18,19,37] (Supplementary Table S1.3). For PCA analysis and differential abundance analysis, the proteinGroups output table from MaxQuant was filtered for reverse database hits, common contaminants, and hits only identified by modified peptides. Then, LFQ intensities were log2 transformed, and replicate samples were grouped. Proteins with less than two valid values in at least one group were removed, and missing values were imputed from a normal distribution centered around the detection limit (package DEP) [61], leading to a list of 2382 reliably quantified proteins in the experiment, which was used for further data analysis. Comparison of protein abundance between pairs of sample groups (MM vs. CTR) was done using the limma package [62], with an FDR < 0.05 and a fold change >4- or <0.25 used as cut-off values for statistical significance. Functional enrichment analysis of upregulated and downregulated protein hits was performed using DAVID [59]. All GO terms represented by at least three proteins at a 0.05 EASE score cut-off were reported.

A detailed description can be found in the Supplemental Material and Methods.

### 4.12. Inflammatory Assays in THP-1 Differentiated Macrophages (THP1-Mac)

The THP-1 cell line was maintained and differentiated to macrophages (THP1-Mac) with 25 ng/mL PMA (Sigma Aldrich, Burlington, MA, USA) for 48 h, as previously described [10], using EV-depleted FBS. The inflammatory potential of isolated EVs was evaluated in 2.5 × 10^5^ THP1-Mac cells plated in 96-well plates with 200 µL media, by treatments with 5 ug/mL EVs quantified according to total protein for each population, over 24 h. The EV aliquots used for each experiment went through only one freeze-thaw cycle. LPS (100 ng/mL) was used as positive inflammatory control. The CM was recovered and used for cytokine quantification by ELISA, as described above. Three independent experiments using EVs isolated from three different experiments, each with triplicates per condition, were performed.

### 4.13. Statistical Analysis

Data are presented as mean ± standard error (SE). Student’s *t*-test was used for comparison between two groups. The non-parametric Mann–Whitney U test was performed to confirm the difference in two-group comparison. For more than two groups, the significance was determined using one-way analysis of variance (ANOVA), with comparison between groups by the Dunnett test. Statistical significance was defined as *p* ≤ 0.05 (*), *p* ≤ 0.01 (**), *p* ≤ 0.001 (***), and *p* ≤ 0.0001 (****).

## 5. Conclusions

Overall, we show that osteogenic VSMCs secrete different populations of EVs with specific protein cargo, adding complexity to the interaction between EVs from different cells usually present in calcified vascular tissues. We highlight the importance of calcifying EVs deposited in the ECM as a new signaling axis in the calcification–inflammation vicious cycle associated with vascular calcification. In these processes, GRP is a clear player involved in the link between osteogenic VSMC differentiation, EV calcification potential, and EMC-EV immunomodulatory properties.

## 6. Patents

The tools and methods described in this manuscript are included in a PCT patent application PCT/PT2009000046.

## Data Availability

The mass spectrometry proteomics data are presented as supplementary material and have been deposited to the ProteomeXchange Consortium/PRIDE repository with the dataset identifier PXD044045 and 10.6019/PXD044045, under the project name: Proteomic characterization of extracellular vesicles (EVs) released by primary human vascular smooth muscle cells (VSMCs) under calcification stress. Reviewer account details: Username: review-er_pxd044045@ebi.ac.uk; Password: i0YPZtpB.

## Supplementary Materials

The following supporting information can be downloaded at: https://www.mdpi.com/article/10.3390/ijms252212406/s1.
