# Supplementary material for: Gla Rich Protein (GRP) Mediates Vascular Smooth Muscle Cell (VSMC) Osteogenic Differentiation, Extracellular Vesicle (EV) Calcification Propensity, and Immunomodulatory Properties
Source: Int J Mol Sci. 2024 Nov 19;25(22):12406. doi: 10.3390/ijms252212406 (PMC11594964; doi:10.3390/ijms252212406)
Supplement: Supplementary file 1 [file ijms-25-12406-s001.zip › Supplementary Material.pdf]

Gla rich protein (GRP) mediate VSMCs osteogenic differentiation, extracellular vesicles (EVs) calcification propensity and immunomodulatory properties

International Journal of Molecular Sciences

Carla Viegas*, Joana Carreira, Teresa Maia, Anjos Macedo, António Pedro Matos, José Neves, Dina Simes

* Corresponding author:
Carla Viegas, Centre of Marine Sciences (CCMAR/CIMAR LA), Universidade do Algarve, Campus de Gambelas, 8005-139 Faro, Portugal
Tel: +351 289 800900; E-mail: caviegas@ualg.pt; ORCID ID: 0000-0002-5765-3665

Supplementary Figures

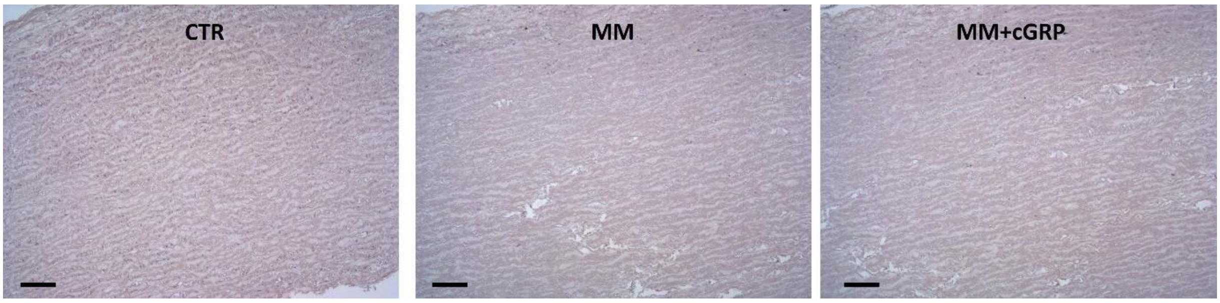

Figure S1: Representative negative controls in consecutive tissue sections of aortic fragments cultured for 12 days in control (CTR), MM and MM supplemented with cGRP (MM+cGRP) conditions, performed by omitting primary antibodies, and counterstaining with HE. Scale bar represents 100 µm.

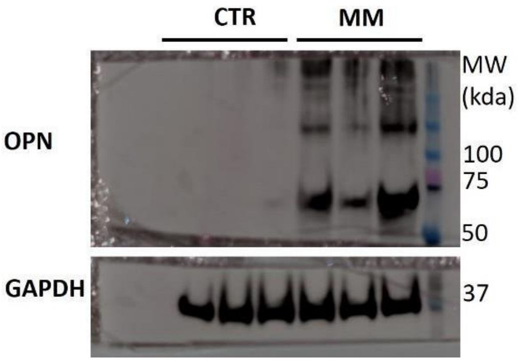

Figure S2: Original membranes for osteopontin (OPN) detection and glyceraldehyde 3-phosphate dehydrogenase (GAPDH) as loading control by western blot, presented as chemiluminescence plus colorimetric images for visualization of membrane edges. Membrane was cut according to molecular weight markers for detection with both antibodies from the same samples. Relevant molecular weight markers (MW) are shown on the right side.

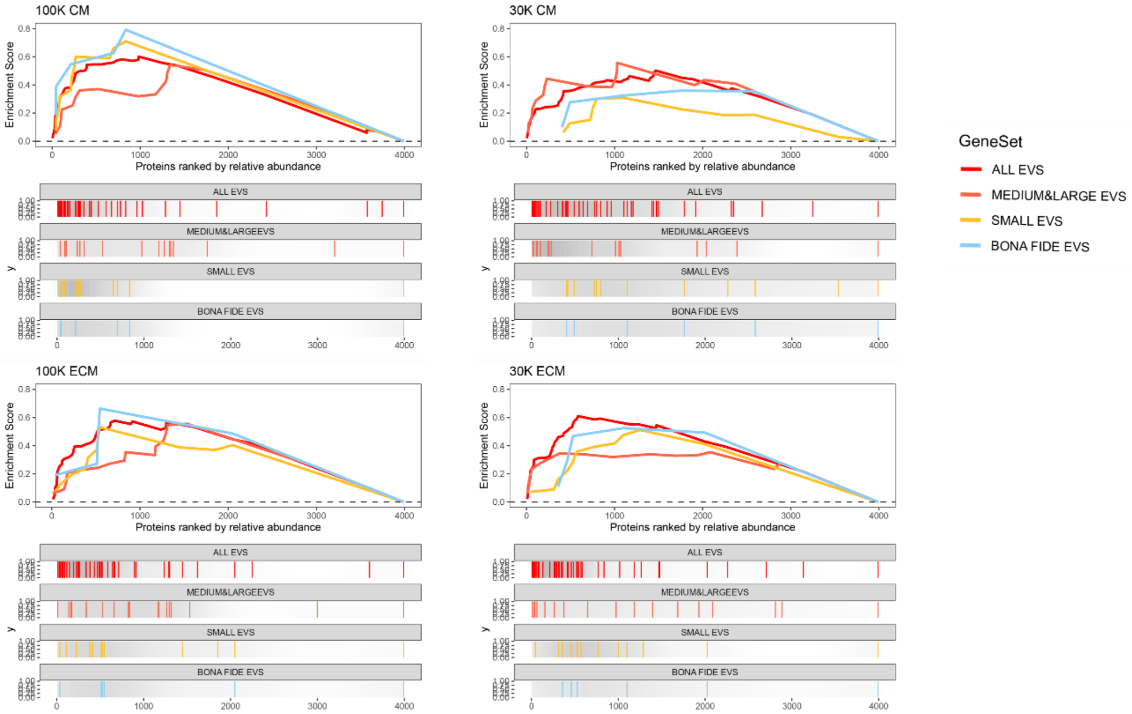

Figure S3: Functional analysis of VSMCs EVs. a. Gene set enrichment analysis performed for each of the EV sample types 100K CM, 30K CM, 100K ECM and 30K ECM to test for their enrichment in bonafide EV markers and in distinct populations of EVs. Protein relative abundances were used as ranking metrics.

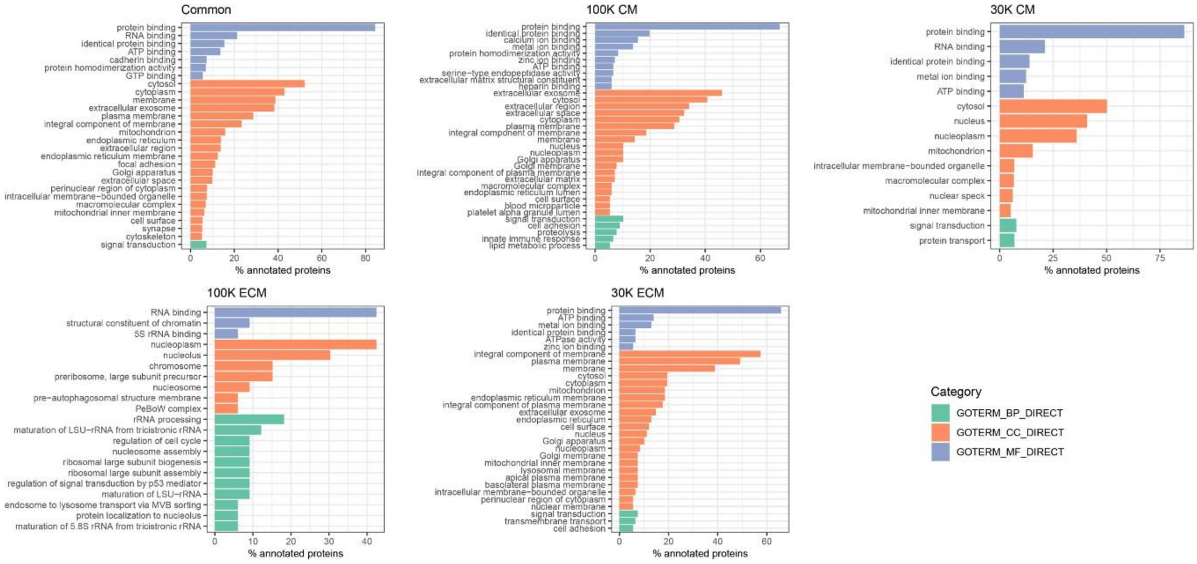

Figure S4: Functional analysis of VSMCs EVs. a. Bar plots showing GO terms enriched (EASE SCORE < 0.05) in the list of proteins commonly identified in all four EV sample types (“Common”) and in the lists of proteins that were specific to the 100K CM, the 30K CM, the 100K ECM or the 30K ECM EV population.

Supplementary Tables

Table S2: Gene-specific primers used in this study

Gene name	Primer name	Sequence (5’ to 3’)
RUNX2	RUNX2_F	TCCGCAGGTCACTACCAGCCACC
	RUNX2_R	GGTGTCACTGTGCTGAAGAGGCTGT
OSX	OSX_F	CAAGGTGTATGGCAAGGCTTCGCA
	OSX_R	TGCTGGCGAGGCAGAAGGTCGGGGCGT
ANAX6	ANAX6_F	GGTGGTGGGAAGGATAGTAAATGT
	ANAX6_R	GGGGCTGGCCTAAGGTCAGAAACA
CASP3	CASP3_F	CAGCTGGTTGGCGTCGCCTTG
	CASP3_R	GCAGGGCACACCCACCGAAAA
GAPDH	GAPDH_F	AAGGTGAAGGTCGGAGTCAACGGA
	GAPDH_R	TCGCTCCTGGAAGATGGTGATGGG
GRP	GRP_F	GTCCCCCAAGTCCCGAGATGAGG
	GRP_R	CCTCCACGAAGTTCTCAAATTCATTCC
MGP	MGP_F	TGGAGGCTGGCACCTGATTTTG
	MGP_R	AAAAGGGGTGCAGCCAGACAAG
ASMA	ASMA_F	TGGCTATTCCTTCGTTACTACTGCTGAG
	ASMA_R	GCCCATCAGGCAACTCGTAACTCT
OPN	OPN_F	ACGGACCTGCCAGCAACCGAAGT
	OPN_R	TACTGGATGTCAGGTCTGCGAAA
TNFA	TNFA_F	AGGGCCTGTACCTCATCTACTCCCA
	TNFA_R	AGCTGGAAGACCCCTCCCAGATAGA
IL1B	IL1B_F	TGGACAAGCTGAGGAAGATGCTGGT
	IL1B_R	CCCTGGAGGTGGAGAGCTTTCAGTT
